# Perception of Bullying in Early Childhood Education in Spain: Pre-School Teachers vs. Psychologists

**DOI:** 10.3390/ejihpe13030050

**Published:** 2023-03-20

**Authors:** María-Luz Fernández-Alfaraz, María Nieto-Sobrino, Álvaro Antón-Sancho, Diego Vergara

**Affiliations:** Technology, Instruction and Design in Engineering and Education Research Group, Catholic University of Ávila, C/Canteros s/n, 05005 Ávila, Spain; mluz.fernandez@ucavila.es (M.-L.F.-A.); maria.nieto@ucavila.es (M.N.-S.); alvaro.anton@ucavila.es (Á.A.-S.)

**Keywords:** bullying, harassment, violence, school, survey

## Abstract

In this work, quantitative research is carried out on the perceptions of early childhood education teachers and child psychologists about the incidence of bullying behaviors in early childhood education (3 to 6 years old) and their knowledge and experience in this regard. For this purpose, two questionnaires were used, each of them oriented to one of the two groups of professionals analyzed, whose answers were subjected to statistical analysis. As for results, it has been obtained that early childhood education teachers express having received deficient training in school bullying and have little experience in its detection and treatment. Furthermore, the teachers’ conception of bullying occurring in their own schools is not realistic and differs from the knowledge they have of the existence of bullying behavior in other schools. Likewise, the existence of action plans against bullying in the school conditions the teachers’ assessments. The assessments held by psychologists differ significantly from those of teachers, mainly in the identification of the origin of bullying (which teachers attribute to the use of digital technologies and psychologists to the social and family environment of the aggressor). Finally, some implications and recommendations in terms of the training of educational professionals on bullying and the need for greater collaboration between teachers and psychologists are reported.

## 1. Introduction

Bullying is a problem consisting of the occurrence of repetitive violent behaviors of one or more students towards one or more others, and its existence is widespread throughout the world [1,2]. Thus, isolated violent or hostile acts are not considered bullying, but for bullying to occur, there must be behaviors that occur repeatedly and reflect the inequality of power between the participants [2,3]. Bullying leaves serious social consequences at the educational and health levels [3], especially affecting the victims [3,4,5]. It is a growing phenomenon that affects students at all educational stages, from early childhood, and that transcends the limits of physical presence, becoming visible in environments outside the school, such as social networks or online games [6,7]. Although the most visible cases of bullying occur in second childhood (from 6 to 12 years) and adolescence (from 12 to 18 years), this phenomenon also seems to have a significant presence in the early childhood education stage (from 3 to 6 years). In fact, the young age of the agents involved in bullying at this stage constitutes one of the most aggravating factors of this phenomenon [5,8], since there is a certain tendency to erroneously minimize the importance and seriousness of bullying situations by teachers, precisely because of the young age of the children involved [9,10]. Along this line, another aggravating factor that hinders the early diagnosis of the possible presence of bullying in an educational center is the silence that both victims and witnesses of bullying tend to keep in the face of these situations because of the fear they feel [4,9]. This highlights the need to provide comprehensive and specific training to pre-service teachers in the detection of bullying in order to overcome these difficulties [11,12].

It is also worth noting the importance of the anti-bullying action plans that some educational centers have in place. These plans make it possible to carry out a sociological study of the student body to detect at an earlier stage any type of hostile situation among students [13], as well as the possible conditioning factors that may predispose a person to being a victim, such as unpopularity, shyness, educational needs, ethnicity or sexual orientation. Likewise, it would be observed whether a student could be a possible aggressor or have a role as a “student mediator” who would help the victim by informing teachers about any conflict inside or outside the classroom [1,2,14]. There are several methods to control bullying, which are chosen by schools according to their preferences and needs. It has been shown that the presence of anti-bullying action plans, such as mediation systems among students, decreases the incidence of bullying, as well as the possible sequelae of victims in those centers that have them [15,16,17].

Another aspect that makes it difficult to identify bullying situations in early childhood education is the diversity of forms that bullying can take. In this sense, studies focused on the analysis of the concentrated locations of harassment show that the most interesting scenario is the school playgrounds [18]. In them, bullying situations derived from the pressure to demonstrate more power among peers are observed [19,20]. In this sense, it is necessary to describe the personality of the aggressors, who may present diverse characteristics that may include aggressiveness towards the rest of the students and even adults, leadership charisma and strong temperament, observing in most cases an absence of empathy towards the victim [21,22]. The literature also identifies bullying scenarios far from the educational center, reporting bullying behaviors experienced by minors in their homes or social and cultural environments [23,24,25]. These aspects can trigger the development of aggressive behaviors among children derived from possible exposure to hostile behavior or peer pressure [19,26]. Added to this is the emergence today of other dangers arising from increased exposure to online environments [26,27] that child psychologists describe as triggering defiant and hostile attitudes among minors [28,29].

It has been shown that the attitude presented by teachers to possible bullying situations can determine the roles of those involved in them [30]. This highlights the need to know the perceptions that professionals in the educational field have about bullying. In this regard, it is useful to differentiate between professionals in early childhood education (children under 6 years of age) and those in primary education (children aged 6 to 12 years). Indeed, although bullying situations that appear in early childhood education can extend to the primary stage, in the latter, there are some triggers that are infrequent in the former, such as popularity or social status, which make it necessary to distinguish between the two stages [31,32].

The literature reveals that the incidence of bullying, regardless of the educational stage, is unequal in different geographical regions. Thus, there are countries such as Spain, on which this study focuses, whose incidence of bullying is very high [11], especially towards students with educational needs [33]. The phenomenon of school bullying in Spain occurs both in urban areas and in rural areas with low population density, such as the region of Castilla y León, which is the place of origin of the participants in the present study [34]. These studies reflect the lack of confidence on the part of teachers to identify and, where appropriate, alleviate bullying situations in the classroom and even a certain tendency of teachers to underestimate bullying situations in the classroom, attributing them to children’s attitudes, especially in the infant stage [35]. In addition, the role of psychologists or psychopedagogues in educational centers should also be highlighted, who help victims to deal with their emotional and behavioral aspects, as well as to promote and carry out mediation plans to improve academic performance in cases of bullying [36]. 

From all the above follows the need to analyze the perception of teachers and psychologists about bullying and the urgency for schools to design and implement specific plans against bullying to alleviate any type of hostile behavior present in the school [11,37,38,39]. Therefore, the aim of this research is to analyze the perception of early childhood education teachers and psychologists regarding this problem and the training they present in this regard, as well as the management of possible support provided by the school to victims and their families [40,41]. The greater aim is to discuss the training needs of the educational and social community in the field of bullying so that this will help to improve the measures for detection and action against bullying in schools, thus minimizing the possible sequelae of minors who present them [42].

## 2. Materials and Methods

### 2.1. Participants

**Figure 1:**
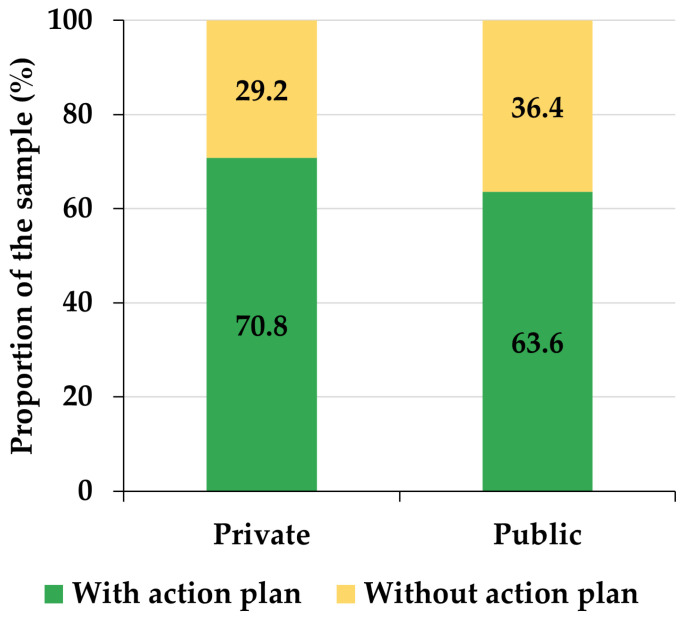
Distribution of participating teachers by school tenure and according to whether their school has an action plan against bullying.

Two samples of early childhood education professionals participated in the study: one formed by early childhood education teachers and the other formed by child psychologists. On the one hand, the first sample is made up of 70 early childhood education teachers, all of them female, with a mean age of 39.14 years (sd = 8.87) and a median age of 40 years, chosen by a non-probabilistic sampling process by convenience among the authors’ contacts. All early childhood education teachers are university graduates in Spanish early childhood education. A total of 68.57% belonged to private schools, while 31.43% came from public schools, which implies that there is a certain bias in favor of private schools (chi-square = 4.8286, df = 1, *p*-value = 0.0280). Likewise, 68.57% of the participating teachers have an action plan against bullying in their respective centers, while the remaining 31.43% do not. In this sense, there is also a certain bias in favor of teachers whose schools have an anti-bullying plan (chi-square = 4.8286, df = 1, *p*-value = 0.0280). The distribution of participants in schools with or without an anti-bullying plan is statistically independent of the way they are distributed in private or public schools (chi-square = 0.1813, df = 1, *p*-value = 0.6703) (Figure 1). Private early childhood education schools usually develop a specific educational project, which is that of its own entity. This owning entity is often, but not necessarily, a religious entity. Likewise, the families who take their children to these private schools do not necessarily belong to the economically privileged strata of society, because, for the most part, these schools are partially supported by Spanish public funds. In addition, it is worth noting that the number of pupils in Spanish pre-school education tends to range between 15 and 25 in each class. Moreover, the difference between average class sizes in public and private pre-primary schools is not significant in Spain.

On the other hand, the sample of psychologists is made up of 16 professionals (10 males and 6 females) chosen through a non-probabilistic convenience sampling process among the authors’ contacts within the region of Castilla y León, in Spain. All of them, except one, have dealt with cases of bullying. Although the sample size is small, this is due to the high specificity of the professionals surveyed (early childhood psychologists), who are not abundant in absolute terms.

### 2.2. Objectives and Variables

The general objective of the present research is to analyze the perception of early childhood education teachers and child psychologists about the situation of bullying in the early childhood education stage (3 to 6 years of age). Specifically, the following research objectives are sought: (i) to describe the perception that early childhood education teachers have about the training they have received about bullying, how it is living together in their educational center and about the reasons they attribute to bullying; (ii) to describe the awareness that early childhood education teachers have of having seen bullying factors or cases of bullying in their own center and in other educational centers; (iii) to analyze the dependence of the above perceptions on the type of educational center—private or public—of the teacher or on whether the center has a specific action plan against bullying; (iv) to describe the reasons that psychologists attribute to bullying in early childhood education, the reasons why victims of bullying tend to hide their situation, the frequency of different bullying factors in early childhood education and the possibilities that a 6-year-old child victim of bullying has to recover from a psychological point of view; and (v) to compare the perceptions of early childhood education teachers and child psychologists regarding the reasons that each of them attribute to aggressive and bullying behaviors.

**Figure 2:**
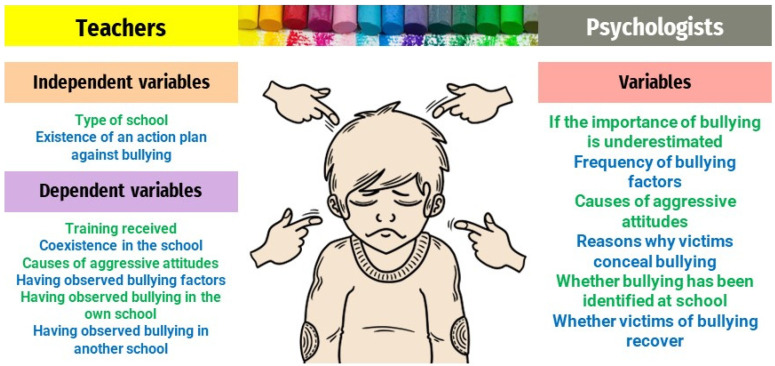
Research variables.

To achieve these objectives, a family of independent and dependent variables has been defined for each of the two populations—teachers and psychologists—studied. Thus, for the population of early childhood education teachers, the following independent variables are considered (Figure 2):

- Type of center: nominal dichotomous variable (private/public);
- Whether the school has a plan of action against bullying: nominal dichotomous variable (yes/no).

The following dependent variables are defined within the population of early childhood education teachers:

- (T1) Training received on bullying: ordinal quantitative variable, measured on a 1 to 5 Likert scale (1—none; 2—insufficient; 3—sufficient; 4—adequate; 5—high);
- (T2) Perception of social interaction in the educational center: ordinal quantitative variable, measured on a 1 to 5 Likert scale (1—very bad; 2—bad; 3—normal; 4—good; 5—very good);
- (T3) Assessment of the influence exerted by the different dimensions of the child’s life—parents, other children, teachers, television and digital technologies—on the possibility of aggressive behavior: ordinal quantitative variable, measured on a 1 to 5 Likert scale (1—null; 2—low; 3—intermediate; 4—high; 5—very high);
- (T4) Awareness of having observed bullying factors: nominal dichotomous variable (yes/no);
- (T5) Knowledge of the existence of bullying situations in early childhood education within the educational center where he/she teaches: nominal dichotomous variable (yes/no);
- (T6) Knowledge of the existence of bullying situations in early childhood education in a school other than the one in which he/she teaches: non-minimal dichotomous variable (yes/no).

Within the population of child psychologists, the following research variables were defined for the population of psychologists (Figure 2):

- (P1) Perception of whether the importance of bullying situations is underestimated in the childhood stage: nominal dichotomous variable (yes/no);
- (P2) Assessment of the frequency with which the different bullying factors occur in early childhood education: ordinal quantitative variable, measured on a 1 to 5 Likert scale (1—never; 2—rarely; 3—occasionally; 4—frequently; 5—very frequently);
- (P3) Assessment of the influence exerted by the different dimensions of the child’s life—parents, other children, teachers, television and digital technologies—on the possibility of aggressive behavior: ordinal quantitative variable, measured on a 1 to 5 Likert scale (1—null; 2—low; 3—intermediate; 4—high; 5—very high);
- (P4) Assessment of the reasons why children in early childhood education who are victims of bullying hide their situation: ordinal quantitative variable measured on a 1 to 5 Likert scale (1—almost never; 2—not usually; 3—occasionally; 4—usually; 5—almost always);
- (P5) Knowledge of the existence of bullying situations in early childhood education in the educational center where he/she teaches: nominal dichotomous variable (yes/no);
- (P6) Perception of whether victims of bullying in early childhood education recover psychologically: dichotomous nominal variable (yes/no).

### 2.3. Instrument

For this research, two questionnaires were used, one for each of the target populations—teachers and psychologists—of the study. The questions of the questionnaires, as well as their link with the dependent variables that were defined, are shown in Table 1, for the early childhood education teachers, and in Table 2, for the psychologists.

From the computation of Cronbach’s alpha parameter, it can be deduced that both the family of questions corresponding to variable T3 (α = 0.9031) and that corresponding to variable P3 (α = 0.8163) enjoy high levels of internal consistency.

### 2.4. Methodology and Statistical Analysis

**Figure 3:**
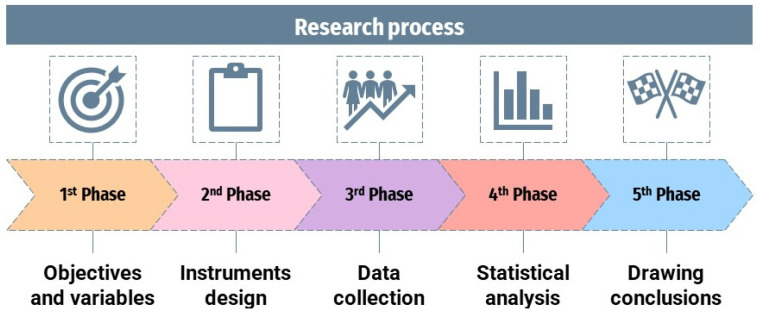
Research phases.

In this paper, we conducted descriptive quantitative research on the perception of early childhood education teachers and child psychologists about the situation of bullying in the early childhood education stage (3 to 6 years of age). For this purpose, the following research phases were followed (Figure 3): (i) definition of research objectives and variables; (ii) design of questionnaires; (iii) data collection; (iv) statistical analysis; and (v) drawing of conclusions. 

Two families of responses were obtained for the two questionnaires used as research instruments, which were applied to each of the samples of professionals—teachers and psychologists—with whom we worked. The statistical analysis carried out with the teachers’ answers follows the following steps:

1. The computation of descriptive statistics for the different dependent variables T1 to T6 and the identification of significant differences between the different factors identified as influencing the development of bullying behaviors (T3) using the Kruskal–Wallis nonparametric test;
2. Analysis of the dependence of variables T1 to T4 on the values of the dependent variables—type of school and existence of an action plan against bullying in the school. For this purpose, we used the bilateral Wilcoxon test for comparison of means for independent samples—variables T1 to T3—or Pearson’s chi-square test of independence for variable T4;
3. Analysis of the dependence of variables T5 and T6 on each other, in order to analyze whether working in an educational center influences the acquisition of awareness of cases of bullying occurring in the center itself. For this purpose, Pearson’s chi-square test of independence was used;
4. Analysis of the dependence of variables T5 and T6 on the dependent variables using Pearson’s chi-square test of independence.

For the analysis of the answers given by the psychologists to their respective questionnaires, the following steps were followed:

1. The computation of descriptive statistics for the different dependent variables P1 to P6 and the identification of significant differences between the different factors identified as influencing the development of bullying behaviors P3 using the Kruskal–Wallis nonparametric test;
2. Comparison of the mean ratings of the degree of influence of the different dimensions of the child’s life on the development of aggressive attitudes between teachers and psychologists. For this purpose, the bilateral Wilcoxon test was used to compare the means of the responses of the variables T3 and P3.

## 3. Results

### 3.1. Responses of Teachers

The participating teachers gave a low rating to the training they have received on bullying, but the rating of social interaction at school was very good, with low dispersion and negative asymmetry, indicating a high frequency of rating 5, which is the highest (Table 3). The factors perceived as most strongly influencing the development of bullying attitudes are television and the use of networks and digital technologies, although, in any case, the influence is considered moderate—around 4 out of 5. The influence that participants attribute to social networks and television on the development of bullying behaviors is due to the frequency with which children see violent scenes through them and the fact that violence is often normalized in these media. The influence of parents and other children is considered low and that of teachers very low—below 2 out of 5. In the case of the influence of teachers, this is the one with the highest deviation and coefficient of variation, and a high positive skewness, which indicates a strong choice of option 1—null influence of teachers—by the participants. From the Kruskal–Wallis test statistics applied to the different influence factors analyzed in variable T3, it follows that the superiority of the ratings of technologies and television to the detriment of the other options is statistically significant (chi-square = 55.68, df = 4, *p*-value < 0.0001). 

There are no significant differences between private and public schools in the teachers’ evaluations of the training received or social interaction in the school, although in both cases there is a slight superiority, in general, in the evaluations of private school teachers (Table 4). However, the nonparametric Wilcoxon test identifies that there are statistically significant differences between private and public schools in terms of the level of influence exerted on the development of bullying behavior that teachers attribute to each of the factors analyzed (Table 4). In both private and public schools, teachers rate new technologies and television as the main influential factors in the development of bullying behaviors, but the choice of these factors is more frequent among teachers in private schools. Teachers in public schools, on the other hand, value to a lesser extent than their colleagues in public schools the influence of parents or other children in the environment (Table 4). 

Finally, teachers whose schools have an action plan against bullying report having received better training on bullying and rate social interaction in their schools more highly than those whose schools do not have such an action plan, the latter difference being statistically significant (Table 5). Likewise, teachers whose schools have an anti-bullying plan value to a greater extent the influence of parents, teachers and other children and less the influence of new technologies and television than those whose schools do not have an anti-bullying plan, although the Wilcoxon test does not report statistical significance in these differences (Table 5).

**Figure 4:**
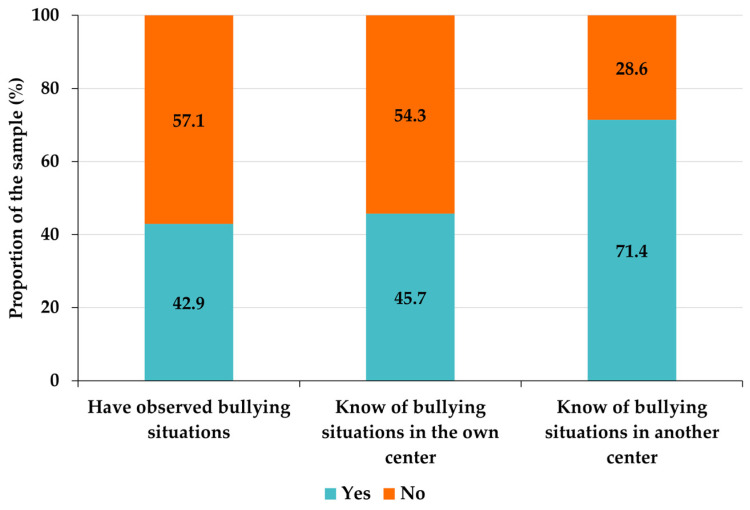
Proportions of the responses to the questions corresponding to variables T4 to T6—awareness of having observed bullying factors and knowledge of the existence of bullying situations in their own center as well as in other centers.

Teachers are distributed approximately homogeneously among those who have observed bullying situations in early childhood education and those who have not (chi-square = 1.4286, df = 1, *p*-value = 0.2320). Likewise, the proportion of those who know of bullying situations occurring in the center itself is approximately similar to that of those who do not (chi-square = 0.5143, df = 1, *p*-value = 0.4733). However, the proportion of those who are aware of bullying situations in schools other than their own is almost three times that of those who are not (Figure 4), the difference between the two proportions being statistically significant (chi-square = 12.8570, df = 1, *p*-value = 0.0003).

Among the teachers who are aware of bullying situations in their own school, a large majority are teachers who are also aware of bullying situations in other schools (87.5%, compared to 12.5% who are not aware of bullying situations in other schools). However, among teachers who are not aware of bullying situations in their own school, slightly more than half are aware of bullying situations in other schools (57.9% are aware of bullying situations in other schools, compared to 42.1% who are not). The distribution between affirmative and negative responses for the two questions is not independent, as shown by Pearson’s chi-square test of independence statistics (chi-square = 7.4605, df = 1, *p*-value = 0.0063). Consequently, more than half of those who do not know about bullying situations in their own school do know about them in other schools, and almost 90% of those who know about bullying situations in their own school also know about them in other schools, these distributions being statistically significant.

The proportion of teachers who have not witnessed bullying situations is higher than that of those who have witnessed it in both private and public schools, but the gap between negative and affirmative responses is greater among teachers in public schools (Table 6). Likewise, in both types of schools, there is a higher proportion of teachers who do not know of bullying situations in their own school than those who do, but there are more who know of bullying situations in other schools than those who do not. The gaps are larger, again, in public schools than in private schools (Table 6). 

In private schools, there is a slight majority of teachers who are aware of bullying situations in their own school, while in public schools, the number of teachers who are not aware of bullying in their own school is almost three times higher than those who are aware of it, and this gap is statistically significant (Table 6). Teachers working in schools that do not have a specific plan of action against bullying observe more bullying situations in their own schools than those whose school has a plan against bullying, and they are also aware of cases of bullying in other schools in a higher proportion than the latter (Table 7). However, Pearson’s test of independence does not allow us to assume that these differences are significant.

### 3.2. Responses of Psychologists

A total of 62.5% of the psychologists surveyed believe that, in general, the seriousness of the problem of bullying in early childhood education is underestimated, compared to 37.5% who believe that it is not underestimated. The participating psychologists are familiar with bullying situations because they have dealt with these types of cases in higher educational stages (primary and secondary). It should be highlighted here that many of the participating psychologists, despite not having dealt with children under 6 years of age, have been able to intuit in their diagnoses with primary school children that some of the bullying situations of such children could have started in the early childhood stage. All of them believe that having suffered bullying in childhood leaves persistent sequelae. As for the forms of bullying observed in early childhood education, the most frequent, in the opinions of the participating psychologists, are teasing, rejection and threats, which are also the ones with the lowest rates of variation. The least frequent are theft and aggression, although with higher rates of variation (Table 8).

As for the factors that influence the development of bullying attitudes, the psychologists mostly identify the influence exerted by parents and other children in the environment, above that exerted using new technologies or television (Table 9). The least influential factor, from the perspective of the participating psychologists, is the influence of teachers. The responses on the influence of parents, other children and teachers are, moreover, those with the lowest rates of variation, which means that they are the ones with the greatest consensus. The differences between the ratings of the factors considered are statistically significant, as can be deduced from the Kruskall–Wallis test (chi-square = 13.271, df = 4, *p*-value = 0.0100).

As for the reasons why child victims of bullying tend to hide the situation they experience, psychologists believe that the main reasons are fear of reprisals and shame (Table 10). These responses are also given with the lowest deviations and rates of variation, which shows that there is a strong consensus among the participants in this regard.

### 3.3. Comparison of Teachers and Psychologists

**Figure 5:**
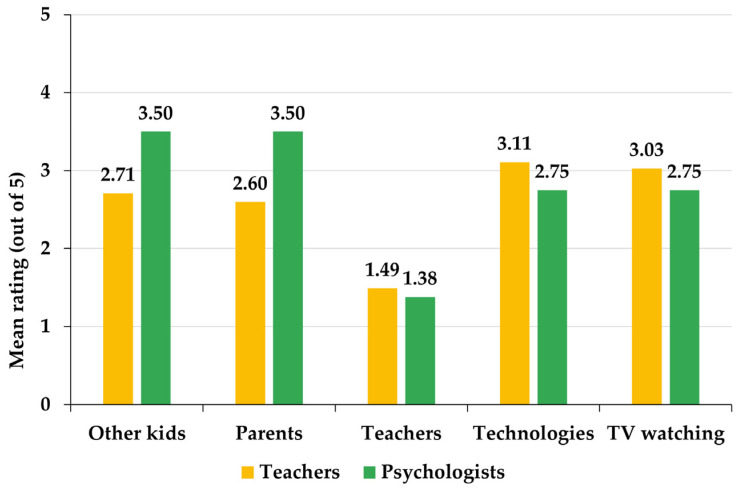
Comparison of the mean responses of teachers and psychologists to the questions corresponding to variables T3 and P3—assessment of the influence exerted by the different dimensions of the child’s life.

Teachers and psychologists agree that the influence of teachers is the least determinant factor in the development of bullying behavior. However, teachers believe that the most influential factors are the use of new technologies and television, while psychologists favor the influence exerted by parents and other children in the environment (Figure 5). In this sense, the greatest distances between psychologists and teachers are precisely in the evaluations of the influence of parents and other children, which are valued, respectively, 34.62% and 29.15% more by psychologists than by teachers.

## 4. Discussion

The participating early childhood education teachers rated the training they had received on bullying as insufficient (Table 3). The deficient training of teachers on bullying [34,35] and the insufficiency of specific teacher training plans on this topic [37] are aspects abundantly pointed out in the literature [38,39]. The quality of teacher training is crucial to the design of anti-bullying action plans and their effective implementation [12], so the insufficient training revealed by the results may explain, at least in part, the low rates of schools represented in this study that have anti-bullying action plans.

There are some results obtained about teachers’ ratings that are surprising: (i) on the one hand, social interaction in the school is generally rated as very good (Table 3), but, on the other hand, almost half of the teachers have observed or know of bullying situations within their own school (Figure 4); (ii) there is a disproportion between teachers who know of the existence of bullying situations in their own school—slightly less than half—and teachers who know of bullying situations in other schools—almost three-quarters of the total sample. Both results could be explained by the tendency of education professionals to underestimate the importance of bullying situations in early childhood education, an aspect expressed by most of the participating child psychologists (62.5%) and by the previous literature [9]. In fact, the literature shows that early childhood education teachers have a confused and partial concept of what bullying is and only recognize as bullying actions involving physical aggression [10]. This last observation is consistent with the opinions of psychologists, who believe verbal, rather than physical, aggression to be the most common in early childhood education (Table 8), so a concept of bullying restricted to physical aggression would lead teachers not to classify as bullying attitudes and actions that are bullying. In any case, the results suggest the need to complement the perceptions expressed by teachers with research using some of the validated instruments to measure real social interaction in schools [13].

As for the disproportion between knowledge of bullying in the school itself and in other schools, this gap is smaller in schools that have an action plan against bullying (Table 7) and in private schools, which are the most aware of teacher training on bullying (Table 6). It follows that specific training and the existence of an anti-bullying plan lead to greater realism in the identification of bullying situations in early childhood education by teachers. This result is in line with other results found in the literature in the field of primary and secondary education [16,17] but constitutes an original contribution of the present study in terms of its finding in early childhood education.

The factors identified by teachers as the most influential, in general, for the development of aggressive behavior in children in early childhood education are the use of new technologies and television (Table 3). However, among teachers in schools with an anti-bullying plan, the importance given to these factors is similar to that given to the influence of parents and other children (Table 5), which are precisely the most influential factors in the development of aggressive behavior, according to psychologists (Table 9). The preceding literature identifies that the appearance of violent behavior in primary and secondary school children is influenced both by the media and by the existence of aggressive behavior in the domestic environment and in groups of friends [23]. Psychological publications are more likely to identify triggers of aggressive attitudes that have to do with the bully’s family or social context [25]. On the other hand, there are works focused on analyzing the influence that certain specific factors have on the development of bullying attitudes, such as (i) television watching and the excessive use of video games with violent content [28,29]; (ii) parental behaviors of rejection, chaos, coercion or aggressiveness, which favor the development of bullying attitudes and, above all, cyberbullying in young people [27]; (iii) pressure from circles of friends and the social context [19,20]; or (iv) the attitude of teachers, which conditions the adoption of the role of bully or victim by students [30]. As a novel result of the present study, it was found that all these factors are present, in the opinions of teachers and psychologists, in the development of aggressive behavior in early childhood education, but the existence of an action plan against bullying conditions the perception of which factors are considered dominant, which, moreover, are also different among teachers and psychologists (Figure 5).

A problem of concern to psychologists is the tendency of bullying victims to hide the situation they are experiencing, which is mainly attributed to shame and fear of retaliation, according to the psychologists participating in this study (Table 10). However, these results are not completely consistent with the factors indicated by some works in the literature, which identify the perception that victims have of the support available at school and in their immediate environment as the most influential motivation in the decision to remain silent [40]. In contrast, other studies indicate that fear of the reaction of their families is the most decisive factor in this regard [41], which is closer to the results obtained here.

## 5. Limitations and Lines of Future Research

The main limitation of the present study is the size of the sample of teachers and, above all, of child psychologists. The small sample size is explained by the scarcity of registered psychologists practicing their professional work in the geographical region of origin of the participants—official statistics from the Spanish National Institute of Statistics indicate that in Spain, there are only 0.70 psychologists per 1000 inhabitants, a figure that in the region of Castilla y León, where this study is located, drops to 0.23 per 1000 inhabitants, compared to 6.68 physicians and 5.66 nurses per 1000 inhabitants [43]. Despite the proven difficulty of obtaining data from the group of psychologists and the consequent limitation that this fact necessarily introduces in this research, it was decided to include the analysis of the responses obtained from psychologists because the contrast of these responses with those of the population of early childhood education teachers yields novel, original and impactful results for the treatment of bullying in the educational context at the early childhood education stage. However, as a future line of research, it is suggested to compare and contrast the results obtained here with analogous studies carried out in larger samples and spread over larger geographical regions and to complement the quantitative analysis carried out here with other analyses of a qualitative nature.

It is also suggested that the sample of teachers be expanded so that a study analogous to the present one can be carried out in a homogeneous sample by school tenure and by the possession, or the lack thereof, of an action plan against bullying in the center in order to avoid possible biases that could arise from the absence of homogeneity in this respect. In addition, it is also proposed to carry out correlational research to compare the opinions of early childhood education teachers with those of other educational stages in order to identify training and conceptual gaps regarding school bullying among teachers of different stages.

## 6. Conclusions

The training received by Spanish early childhood education teachers on bullying is deficient, which acts as a brake on the implementation and effective execution of action plans against bullying in schools. Likewise, early childhood education teachers have a generally unrealistic perspective on the reality of bullying in the classroom, which is evidenced by the scarce knowledge of bullying situations in their own schools, as opposed to the greater knowledge of bullying situations in other schools, and by the very high evaluation given to social interaction in schools despite the high proportion of teachers who have observed bullying situations. In addition, the above gaps are wider among teachers in whose schools there is no action plan against bullying, so it is suggested that specific anti-bullying plans be implemented in schools, accompanied by commissions to monitor these plans and provide adequate and updated training to teachers.

There are two main gaps between early childhood education teachers and child psychologists regarding the consideration of school bullying: (i) psychologists understand that the most frequent forms of bullying have to do with verbal aggression and rejection, while there is a certain tendency for teachers to not consider these attitudes as bullying and even to underestimate the impact of bullying in early childhood education classrooms; and (ii) psychologists give importance to the child’s social and family environment as decisive factors in developing a bullying role, while teachers consider that the main influential factors in this regard are new technologies and television. It is suggested that early childhood education teachers should be advised by child psychologists working in educational centers more intensively than is currently the case to ensure greater convergence of criteria. It is also recommended that schools and educational administrations hire more psychologists and child mental health specialists to support the detection and treatment of bullying situations in schools and to organize training on bullying for parents and teachers.

## Figures and Tables

**Table 1:** Questions to teachers.

Variable	Item	Question
T1	1	Rate the level of training received on bullying
T2	2	Rate how is the social interaction in your school
T3	3	Assess the level of influence that parents have in the development of bullying behaviors
	4	Assess the level of influence that other children have in the development of bullying behaviors
	5	Assess the level of influence that teachers have in the development of bullying behaviors
	6	Assess the level of influence that television have in the development of bullying behaviors
	7	Assess the level of influence that digital technologies have in the development of bullying behaviors
T4	8	Have you ever observed bullying factors in the early childhood classroom?
T5	9	Have you known about bullying behaviors in the early childhood education classroom within your own school?
T6	10	Have you known about bullying behaviors in the early childhood education classroom in schools other than your own?

**Table 2:** Questions to psychologists.

Variable	Item	Question
P1	1	Assess whether the problem of bullying in early childhood education is usually underestimated
P2	2	Rate the frequency with which peer rejection occurs in early childhood education
	3	Assess the frequency with which threats occur in early childhood education
	4	Rate the frequency with which thefts occur in early childhood education
	5	Assess the frequency with which physical injuries occur in early childhood education
	6	Rate the frequency with which teasing occurs in early childhood education
	7	Rate the frequency with which bullying factors other than the above occur in early childhood education
P3	8	Assess the level of influence that parents have in the development of bullying behaviors
	9	Assess the level of influence that other children have in the development of bullying behaviors
	10	Assess the level of influence that teachers have in the development of bullying behaviors
	11	Assess the level of influence that television have in the development of bullying behaviors
	12	Assess the level of influence that digital technologies have in the development of bullying behaviors
P4	13	Assess the extent to which children who are victims of bullying usually keep silent about their situation for fear of retaliation
	14	Assess the extent to which children who are victims of bullying usually silence their situation out of shame
	15	Assess the extent to which children who are victims of bullying usually keep silent about their situation because they have no one to confide their problem to
	16	Assess the extent to which children who are victims of bullying usually keep silent about their situation because they do not know what to do
P5	17	Do you know of bullying situations in early childhood education?
P6	18	Do you think that children who are victims of bullying in childhood can fully recover psychologically?

**Table 3:** Descriptive statistics of the responses to questions corresponding to variables T1 to T3—training received on bullying, assessment of social interaction in the school and assessment of the influence of the different dimensions of the child’s life.

Question	Mean (Out of 5)	Std. Deviation (Out of 5)	CV	Skewness
Training	2.66	1.14	42.76%	0.48
Social interaction	4.29	0.62	14.51%	−0.27
Influence of other kids	2.71	1.64	60.32%	0.23
Influence of parents	2.60	1.40	53.75%	0.36
Influence of teachers	1.49	1.07	71.85%	2.27
Influence of the use of technologies	3.11	1.66	53.26%	−0.11
Influence of TV watching	3.03	1.50	49.68%	0.00

**Table 4:** Mean responses to the questions corresponding to variables T1 to T3—training received on bullying, assessment of social interaction in the school and assessment of the influence of the different dimensions of the child’s life—differentiating by the type of school and statistics of the *t*-test.

Question	Private	Public	W	*p*-Value
Training	2.71	2.55	572	0.5592
Social interaction	4.33	4.18	600	0.3083
Influence of other kids	3.04	2.00	742	0.0050 *
Influence of parents	2.92	1.91	748	0.0043 *
Influence of teachers	1.67	1.09	642	0.0405 *
Influence of the use of technologies	3.42	2.45	712	0.0164 *
Influence of TV watching	3.33	2.36	736	0.0071 *

* *p* < 0.05.

**Table 5:** Mean responses to the questions corresponding to variables T1 to T3—training received on bullying, assessment of social interaction in the school and assessment of the influence of the different dimensions of the child’s life—differentiating by whether the school has an action plan against bullying and statistics of the Wilcoxon test (bilateral).

Question	With Action Plan	Without Action Plan	W	*p*-Value
Training	2.79	2.36	404	0.0973
Social interaction	4.46	3.91	304	0.0014 *
Influence of other kids	2.42	3.36	692	0.0314 *
Influence of parents	2.71	2.36	456	0.3527
Influence of teachers	1.58	1.27	428	0.0864
Influence of the use of technologies	2.88	3.64	648	0.1182
Influence of TV watching	2.79	3.55	674	0.0590

* *p* < 0.05.

**Table 6:** Proportions of responses (%) to the questions corresponding to variables T4 to T6—awareness of having observed bullying factors and knowledge of the existence of bullying situations in their own center as well as in other centers—differentiating by the school tenure and statistics of the Wilcoxon test (bilateral).

Question	Private		Public		Chi-Square	*p*-Value
	Yes (%)	No (%)	Yes (%)	No (%)		
Have observed bullying situations	45.8	54.2	36.4	63.6	0.5524	0.4573
Know of bullying situations in the own school	54.2	45.8	27.3	72.7	4.3968	0.0360 *
Know of bullying situations in another school	66.7	33.3	81.8	18.2	1.6970	0.1927

* *p* < 0.05.

**Table 7:** Proportions of responses (%) to the questions corresponding to variables T4 to T6—awareness of having observed bullying factors and knowledge of the existence of bullying situations in their own center as well as in other centers—differentiating by whether the school has an action plan against bullying and statistics of the Wilcoxon test (bilateral).

Question	With Plan		Without Plan		Chi-Square	*p*-Value
	Yes (%)	No (%)	Yes (%)	No (%)		
Have observed bullying situations	45.8	54.2	36.4	63.6	0.5524	0.4573
Know of bullying situations in the own school	41.7	58.3	54.5	45.5	1.0083	0.3153
Know of bullying situations in another school	66.7	33.3	81.2	18.2	1.6970	0.1927

**Table 8:** Descriptive statistics of the responses to questions corresponding to variable P2—frequency of occurrence of different bullying factors in early childhood education.

Question	Mean (Out of 5)	Std. Deviation (Out of 5)	CV
Teasing	4.38	1.06	24.24%
Rejection	4.63	0.52	11.19%
Threats	4.00	0.76	18.90%
Theft	2.00	0.93	46.29%
Aggressions	2.75	1.04	37.64%

**Table 9:** Descriptive statistics of the responses to questions corresponding to variable P3—assessment of the influence exerted by the different dimensions of the child’s life.

Question	Mean (Out of 5)	Std. Deviation (Out of 5)	CV
Influence of other kids	3.50	1.41	40.41%
Influence of parents	3.50	1.20	34.15%
Influence of teachers	1.38	0.52	37.64%
Influence of the use of technologies	2.75	1.39	50.50%
Influence of TV watching	2.75	1.16	42.36%

**Table 10:** Descriptive statistics of the responses to questions corresponding to variable P4—assessment of the reasons why children in early childhood education who are victims of bullying hide their situation.

Question	Mean (Out of 5)	Std. Deviation (Out of 5)	CV
Retaliation	4.63	0.52	11.19%
Shame	4.38	0.52	11.83%
Failure to find a reliable person	3.13	1.13	36.03%
Do not know what to do	4.00	1.41	35.36%

## Data Availability

The data are not public. They may be provided upon a reasoned request to the corresponding author.
